# Systemic juvenile idiopathic arthritis is associated with *HLA-DRB1* in Europeans and Americans of European descent

**DOI:** 10.1186/1546-0096-10-S1-A6

**Published:** 2012-07-13

**Authors:** Michael Ombrello, Elaine F Remmers, Alexei A Grom, Wendy Thomson, Alberto Martini, Marco Gattorno, Seza Ozen, Ahmet Gul, John F Bohnsack, Andrew S Zeft, Elizabeth D Mellins, Jane L Park, Claudio Len, Colleen Satorius, Ricardo AG Russo, Terri H Finkel, Rae SM Yeung, Rayfel Schneider, Sampath Prahalad, David N Glass, Roger C Allen, Nico Wulffraat, Pierre Quartier, Maria Odete E Hilario, Kevin Murray, Sheila Oliveira, Jordi Anton, Anne Hinks, Eleftheria Zeggini, Carl Langefeld, Susan Thompson, Jeffrey Chaitow, Justine Ellis, Davinder Singh, Andre Cavalvanti, Blanca Bica, Flavio Sztajnbok, Hakon Hakonarson, Katherine A Siminovitch, Kirsten Minden, Peter Haas, Tobias Schwarz, Daniel L Kastner, Patricia Woo

**Affiliations:** 1 Arthritis Research UK EU, University of Manchester, Manchester, UK; 2 Children's Hospital at Westmead, Westmead, New South Wales, Australia; 3 Children's Hospital of Philadelphia, Philadelphia, PA, USA; 4 Childrens Hospital Medical Center, Cincinnati, OH, USA; 5 Childrens Hospital of PA, Philadelphia, PA, USA; 6 Cincinnati Children's Hospital Medical Center, Cincinnati, OH, USA; 7 Emory Children's Center, Atlanta, GA, USA; 8 Federal University of Rio de Janiero, Rio de Janiero, Brazil; 9 Gaslini Hospital, University of Genoa, Genova, Italy; 10 GCRCA, Garmisch, Garmisch, Germany; 11 German Rheumatism Research Center, Berlin, Germany; 12 Hacettepe University Faculty of Medicine Ankara, Ankara, Turkey; 13 Hopital Necker-Enfants Malades, Paris, France; 14 Hospital for Sick Children, Toronto, ON, Canada; 15 Hospital Sant Joan de Déu, Barcelona, Spain; 16 Hospital Universitário Clementino Fraga Filho – UFRJ, Rio De Janeiro, Brazil; 17 Istanbul University Faculty of Medicine, Istanbul, Turkey; 18 Murdoch Children's Research Institute, Parkville, Victoria, Australia; 19 NHGRI, NIH, Bethesda, MD, USA; 20 Princess Margaret Hospital for Children, Perth, West Australia, Australia; 21 Ricardo Russo, Buenos Aires, Argentina; 22 Royal Childrens Hospital, Melbourne, Australia; 23 Stanford University Medical Center, San Jose, CA, USA; 24 Stanford University Medical Center, Stanford, CA, USA; 25 Toronto General Research Institute, Mount Sinai Hospital, Toronto, ON, Canada; 26 University College London, London, UK; 27 Universidade do Estado do Rio de Janeiro, Rio de Janeiro, Brazil; 28 Universidade Federal de São Paulo, Sao Paulo, Brazil; 29 Universidade Federal de Sao Paulo, Sao Paulo, Brazil; 30 Universidade Federal de Sao Paulo, Sao Paulo, Brazil; 31 University Medical Center Utrecht, Utrecht, Netherlands; 32 University of Utah, Salt Lake City, UT, USA; 33 University of Wuerzburg, Wuerzburg, Germany; 34 Wake Forest University Health Sciences, Winston-Salem, NC, USA; 35 Wellcome Trust Sanger Institute, Oxford, UK

## Purpose

Systemic juvenile idiopathic arthritis (sJIA) is a complex inflammatory disease whose etiology remains unknown. sJIA is distinguished from other forms of juvenile idiopathic arthritis (JIA) by its characteristic features including requisite quotidian fever and salmon-colored, evanescent skin rash, but also by an absence of autoantibodies. Based on its unique phenotype among JIA subtypes, it has been suggested that sJIA may be autoinflammatory rather than autoimmune in nature, and consistent with this, sJIA is distinct among JIA subtypes for its inconsistently detectable association with *HLA* genes. In this study, we sought to use SNP genotyping in a large patient collection to identify sJIA susceptibility loci.

## Methods

We genotyped 576 children fulfilling ILAR criteria for systemic arthritis and 366 control subjects free of sJIA or autoimmune disease. The collection included 205 cases and 210 controls from Cincinnati Children’s Hospital, 185 cases from the repository at University of Manchester, 56 cases and 60 controls from University of Genova, 54 cases from Hacettepe University, 42 cases from the University of Utah, 34 cases from Stanford University, and 96 controls from Istanbul University. SNP genotyping was performed using Illumina Omni1M Quad v1.0 beadchips and iScan platform. Omni1M beadchip data from 60 unrelated CEU HapMap individuals were obtained through Illumina’s iControlDB. SNP associations were evaluated using SNP & Variation Suite 7, excluding SNPs with call rates below 95%, minor allele frequencies below 0.05, or Hardy-Weinberg Equilibrium *p* below 0.001, producing a dataset of 690,672 SNPs in 576 cases and 426 controls. To address population stratification, we employed principal components (PC) analysis to identify and exclude samples with differing genetic backgrounds. We excluded 60 sJIA samples on this basis, reducing the size of the collection for final analysis to 516 cases and 426 controls. After correcting for the top 10 PCs, the genomic inflation factor reflected minimal population stratification (λ_GC_ = 1.01).

## Results

**Figure 1:**
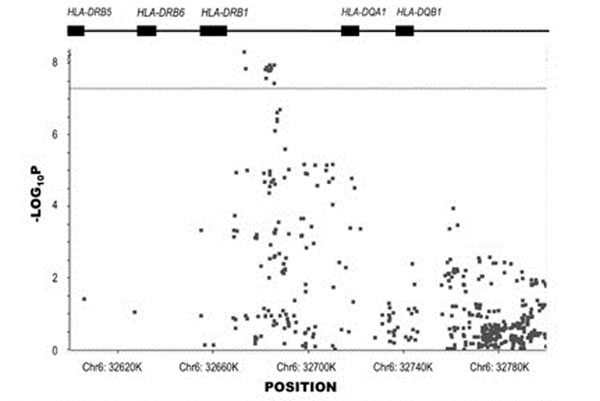
SNP Associations surrounding *HLA-DRB1* in sJIA. Displayed is a plot of the PC-corrected –log_20_p values of association for SNPs surrounding *HLA-DRB1*. Horizontal line at y=7.3 represents genome-wide significance threshold (p < 5x10^-8^).

We identified 12 SNPs within *HLA-DRB1* with PC-corrected associations that exceeded a stringent threshold for genome-wide significance (p < 5 x 10^-8^). These SNPs were part of a larger group of 45 SNPs with *p* < 5 x 10^-5^ in the *MHC* class II gene cluster. The effect size of the sJIA-associated SNPs ranged from odds ratios of 1.45 to 1.65. Notably, the effect size of this association is modest, relative to the effect of associated *HLA* genes in other JIA subtypes and other autoimmune diseases. Figure 1.

## Conclusion

A large collaborative effort to identify sJIA patients and a careful genetic matching strategy have allowed us to clearly detect an association signal within the class II region of the *MHC* of sJIA patients, albeit with more modest effect sizes than those detected in other JIA subtypes. This suggests at least some contribution of autoimmunity to the pathogenesis of this complex disorder.

## Disclosure

Michael Ombrello: None; Elaine F. Remmers: None; Alexei A. Grom: None; Wendy Thomson: None; Alberto Martini: None; Marco Gattorno: None; Seza Ozen: None; Ahmet Gul: None; John F. Bohnsack: None; Andrew S. Zeft: None; Elizabeth D. Mellins: None; Jane L. Park: None; Claudio Len: None; Colleen Satorius: None; Ricardo A.G. Russo: None; Terri H. Finkel: None; Rae S.M. Yeung: None; Rayfel Schneider: None; Sampath Prahalad: None; David N. Glass: None; Roger C. Allen: None; Nico Wulffraat: None; Pierre Quartier: None; Maria Odete E. Hilario: None; Kevin Murray: None; Sheila Oliveira: None; Jordi Anton: None; Anne Hinks: None; Eleftheria Zeggini: None; Carl Langefeld: None; Susan Thompson: None; Jeffrey Chaitow: None; Justine Ellis: None; Davinder Singh: None; Andre Cavalvanti: None; Blanca Bica: None; Flavio Sztajnbok: None; Hakon Hakonarson: None; Katherine A. Siminovitch: None; Kirsten Minden: None; Peter Haas: None; Tobias Schwarz: None; Daniel L. Kastner: None; Patricia Woo: None.

